# Markers of exacerbation severity in chronic obstructive pulmonary disease

**DOI:** 10.1186/1465-9921-7-74

**Published:** 2006-05-10

**Authors:** Luigi G Franciosi, Clive P Page, Bartolome R Celli, Mario Cazzola, Michael J Walker, Meindert Danhof, Klaus F Rabe, Oscar E Della Pasqua

**Affiliations:** 1 Leiden/Amsterdam Center for Drug Research, Leiden University, Leiden, The Netherlands; 2 Sackler Institute of Pulmonary Pharmacology, King's College, London, UK; 3 St. Elizabeth's Hospital, Tufts University, Boston, MA, USA; 4 Department of Respiratory Medicine, A. Cardarelli Hospital, Naples, Italy; 5 Department of Pharmacology & Therapeutics, University of British Columbia, Vancouver, Canada; 6 Department of Pulmonology, Leiden University Medical Center, Leiden, The Netherlands; 7 Clinical Pharmacology & Discovery Medicine, GlaxoSmithKline, Greenford, UK

## Abstract

**Background:**

Patients with chronic obstructive pulmonary disease (COPD) can experience 'exacerbations' of their conditions. An exacerbation is an event defined in terms of subjective descriptors or symptoms, namely dyspnoea, cough and sputum that worsen sufficiently to warrant a change in medical management. There is a need for reliable markers that reflect the pathological mechanisms that underlie exacerbation severity and that can be used as a surrogate to assess treatment effects in clinical studies. Little is known as to how existing study variables and suggested markers change in both the stable and exacerbation phases of COPD. In an attempt to find the best surrogates for exacerbations, we have reviewed the literature to identify which of these markers change in a consistent manner with the severity of the exacerbation event.

**Methods:**

We have searched standard databases between 1966 to July 2004 using major keywords and terms. Studies that provided demographics, spirometry, potential markers, and clear eligibility criteria were included in this study. Central tendencies and dispersions for all the variables and markers reported and collected by us were first tabulated according to sample size and ATS/ERS 2004 Exacerbation Severity Levels I to III criteria. Due to the possible similarity of patients in Levels II and III, the data was also redefined into categories of exacerbations, namely out-patient (Level I) and in-patient (Levels II & III combined). For both approaches, we performed a fixed effect meta-analysis on each of the reported variables.

**Results:**

We included a total of 268 studies reported between 1979 to July 2004. These studies investigated 142,407 patients with COPD. Arterial carbon dioxide tension and breathing rate were statistically different between all levels of exacerbation severity and between in out- and in-patient settings. Most other measures showed weak relationships with either level or setting, or they had insufficient data to permit meta-analysis.

**Conclusion:**

Arterial carbon dioxide and breathing rate varied in a consistent manner with exacerbation severity and patient setting. Many other measures showed weak correlations that should be further explored in future longitudinal studies or assessed using suggested mathematical modelling techniques.

## Background

Chronic obstructive pulmonary disease (COPD) is a respiratory disease characterized by an airflow limitation and inflammation of the lower airways [1]. As the disease worsens, some patients experience 'exacerbations' of their principal symptoms of dyspnoea, cough and sputum. These exacerbations frequently result in a visit to a general practitioner's office or to a local hospital for treatment. Exacerbations occur in COPD patients at a median of three times a year with half of them being unreported [2-4]. The heterogeneity of COPD exacerbations make them difficult to define, classify and manage due to their range of symptoms, varied treatment requirements, seasonal occurrence, and ambiguous aetiology [5-14].

To address this problem, attempts have been made to develop a consensus definition for COPD exacerbations [15]. Recently, the American Thoracic Society (ATS) and European Respiratory Society (ERS) adopted the following definition: *'an event in the natural course of the disease that is characterised by a change in the patient's baseline dyspnea, cough and sputum beyond day-to-day variability sufficient to warrant a change in management' *[1].

The severity of an exacerbation has been also difficult to classify despite the various schemes that have been proposed to deal with this issue [4,15-17]. The ATS and ERS have also jointly suggested a classification based upon severity and the type of medical management used, i.e., Exacerbation Level I is home treatment, Level II is hospitalization, and Level III is specialised care [1]. The aim of this scheme is to improve the existing management of exacerbations and to serve as an aid in the assessment of treatment efficacy.

Different operational definitions for COPD exacerbations have been proposed in the past and these have helped determine their relative importance, in particular their relationship to COPD progression [1-17]. However, these definitions have relied primarily on symptoms, and this along with the absence of a standard classification for the degree of symptom severity, has delayed the development of new therapies for this condition. The current therapies for exacerbations have been evaluated based on their ability to reduce symptoms, and to improve a patient's forced expiratory volume in one second (FEV_1_) since the latter is strongly correlated with COPD mortality. However, FEV_1 _does not discriminate well between the stable and exacerbative states of COPD, particularly during the later stages of this disease. Hence, the development of biological markers, or biomarkers that are more sensitive and specific to the severity of COPD exacerbations would provide investigators with new insights and directions for further research.

At this time, only a few clinical variables or inflammatory mediators have been shown to be associated with COPD exacerbations and their related morbidity and mortality. Some of those include: age [18-20]; FEV_1_, forced vital capacity and peak expired flow [19,21,22]; body mass index [20]; albumin [20,22,23]; sodium [23]; pH [24,25]; eosinophils [26-29]; interleukins 6 and 8 [29-32]; fibrinogen [31]; and C-reactive protein [33]. Significant clinical events such as the number of exacerbations per year, the number of hospital admissions per year, time to relapses, and days in hospital have been regarded as useful measures in clinical studies designed to assess drug efficacy and cost-effectiveness as well as to standardize existing hospital support programs for COPD [34-39]. However, it is not known how these measures change with increasing severity of COPD exacerbations.

Therefore, we have surveyed the medical literature to identify which of the commonly accepted variables and suggested markers for COPD exacerbations change according to the ATS/ERS' levels of exacerbation severity. The long-term aim of our work is to assess the sensitivity and specificity of potential markers for use in future COPD studies as well as to determine how such markers can be further studied and fully integrated into the development of new drugs for COPD.

## Methods

### Search strategy, study selection and overall objectives

We searched standard databases since 1966 using medical search headings and related terms as obtained from major consensus documents related to COPD exacerbations. The major keywords were 'exacerbation', 'unstable', 'acute', 'bronchitis', and variants of the term 'COPD'. This phase of our search retrieved a total of 843 citations. For these citations, we read the title and abstract of each citation so as to exclude citations that concerned exacerbations of coronary artery disease, myocardial infarction, cystic fibrosis, asthma, pulmonary emboli, and community pneumonia. Citations for case studies, letters, reviews, meta-analyses, and animal studies were also excluded.

After this initial screening, we identified 387 citations to papers that were of possible interest. We retrieved the original articles in electronic and hard copy forms, and then critically read each article. As a result of this step, we arrived at a total of 268 studies in our final review and analyses. We selected these studies based on the availability of demographics, spirometry, clear study eligibility criteria, and the potential markers being used to assess exacerbations.

The objectives of this literature review and data analyses were to determine which of the baseline measures commonly used in COPD exacerbation studies change with the extent of the exacerbation and disease severity, and to determine whether COPD exacerbations can be modelled as 'events' or 'time-to-event' in future investigations.

### Data abstraction methods

Initially, we considered various exacerbation definitions and classification schemes, in particular, those suggested by Rodriguez-Roisin [15] as well as those described by Pauwels and colleagues [17]. However, we determined that the ATS/ERS' operational classification of exacerbation severity [1] was the most sensible and feasible system for systematically assessing the patient baseline characteristics and biomarker information from the majority of published studies. We therefore used this classification scheme and the related clinical history, physical findings and diagnostic procedures for managing exacerbations to perform our data abstraction. From each study, we retrieved the reported demographics, spirometry, smoking status, clinical, cytological and biochemical variables as well as suggested markers of the severity of the exacerbation at baseline conditions, i.e., immediately prior to, or during the exacerbation event but before the time in which the intervention of interest was investigated (Table 1). Whenever such variables were measured in stable conditions, we also abstracted this information. For each study, we noted the type of definition used to define an exacerbation such as symptom- or event-based as well as the research question asked, the experimental design used, any sponsorship, and the presence or absence of data from individual study patients. Data was then further organized according to sample size and smoking status when available. Cytological and biochemical data were also classified according to their collection methods. These included sputum induction, bronchial biopsy, bronchoalveolar lavage (BAL), exhaled breath sampling, and blood sampling.

**Table 1:** Commonly Accepted Measures and Potential Markers Associated with COPD Exacerbations

Demographics	Age; Gender; Height; Weight; Body Mass Index (BMI); Disease Years; Pack-Years.
Spirometry/Respiratory Status Measures	Forced Expired Volume in One Second – FEV_1 _(litres and % predicted); Forced Vital Capacity – FVC (litres and % predicted); FEV_1_/FVC Ratio; Breathing Rate; Oxygenation Saturation (pulse and arterial); Peak Expired Flow Rate (PEFR); Fraction of Inspired Oxygen (FiO_2_); Intrinsic PEEP, Arterial Oxygen Tension (PaO_2_); Arterial Carbon Dioxide Tension (PaCO_2_); PaO_2_/FiO_2 _Ratio; Sputum Production.
Dyspnea Measures	Baseline Dyspnea Index (BDI); Translational Dyspnea Index (TDI); Medical Research Council (MRC) Dyspnea Scale; Borg Dyspnea Score; American Thoracic Society (ATS) Dyspnea Score.
Functional Challenge & Quality of Life Measures	6 Minute Walking Distance (6MWD); β_2_-Agonist Reversibility; Adenosine Monophosphate, Methacholine or Histamine Challenge; St. George's Respiratory Questionnaire (SGRQ).
Haemodynamic Measures	Systolic Blood Pressure; Diastolic Blood Pressure; Arterial Blood Pressure; Heart Rate; Cardiac Output.
Electrocardiogram Measures	Lead II; Lead aVF; P Wave Axis-degrees.
Blood Electrolyte, pH and Protein Measures	Sodium; Potassium; Chloride; Bicarbonate; Glucose; pH; Phosphate; Urea; Albumin; Haemoglobin; Creatinine.
Exacerbation-Related Measures	Number of Exacerbations Per Year (or in Past Year); Number of Exacerbations Per Patient-Year; Number of Exacerbations Requiring Oral Corticosteroids Per Patient-Year; Number of Exacerbation Related Infections in Past Year; Days in Hospital; Days Per Patient-Year in Hospital; Days in Intensive Care Unit; Days on Mechanical Ventilation; Number of Unscheduled or Scheduled GP Visits in Past Year; Time to First Exacerbation.
Hospital-Related Measures	Number of Admissions in a Year; Number of Admissions Per Patient-Year; Number of Emergency Department Visits in Past Year; Time in the Emergency Department; Number of Patients Hospitalized in Past Year; Number of Patients Readmitted in Past Year; Number of Patients Relapsed in Past Year; Ventilation Type – Non-Invasive Positive Pressure Ventilation (NIPPV), Invasive Mechanical Ventilation (IMV) or Iron Lung; Admission to ICU; Mortality in Intensive Care Unit; Mortality in Hospital; Simplified Acute Physiology II Score (SAPS II); Acute Physiology And Chronic Health Evaluation II (APACHE II); Glasgow Coma Score (GCS).
Reported Comorbidities – Presence/Absence; Number & Percent of Subjects	Charlson Comorbidity Index; Cardiovascular Disease; Cor Pulmonale; Congestive Heart Failure/Insufficiency; Coronary Heart Disease; Ischaemic Heart Disease; Cardiac Arrhythmia; Hypertension; Pulmonary Oedema; Cerebrovascular Disease; Renal Disease; Liver Disease; Gastrointestinal Disease; Peripheral Vascular Disease; Endocrine Disease; Diabetes Mellitus; Cancer; Deep Vein Thrombosis; Pulmonary Emboli + Deep Vein Thrombosis; Bronchiectasis; Asthma; Depression; Emphysema; Comorbidity Present, Excluded from Study, or Not Described.
Reported Causes of Exacerbations – Presence/Absence; Number & Percent of Subjects	Pneumonia; Sepsis; Bronchospasm; Viral Infection; Bronchial Infection; Infection; Cardiac Insufficiency/Heart Failure; Cardiac Arrhythmia; Pulmonary Emboli; Unknown Cause.
Reported Drug Information – Presence/Absence; Number & Percent of Subjects	Beta Agonists – Inhaled, Short-Acting, Long-Acting, Oral/IV Systemic; Corticosteroids – Inhaled, Oral/IV Systemic; Theophylline; Xanthines; Bronchodilators; Anticholinergics; Long-Term Oxygen Therapy (LTOT); Oxygen Supplementation; Beta-Agonist-Corticosteroid & Beta-Agonist-Anticholinergic Combinations; Antibiotics; Mucolytics; Expectorants; Antitussives; Diuretics; Oral Anticoagulants; Patient Compliance.
Bacterial Information – Number of Patients/Number of Isolates	*S. pneumoniae; H. influenzae; M. catarrhalis; P. aeruginosa; B. catarrhalis; H. parainfluenza; S. aureus; C. pneumoniae; E. coli; *OTHER: *K. pneumoniae, Enterobacteriaceae, Pseudomonas *Species, *Alpha-Haemolytic Streptococci, Acinetobacter, M. pneumoniae, Legionella *Species.
Viral Information – Number of Patients/Number of Isolates	Influenza Virus A & B; Parainfluenza V1, V2 & V3; Adenovirus; Respiratory Syncytial Virus (RSV); Picornavirus; Rhinovirus; Coronavirus.
Cytological Measures – Local (Sputum, Bronchoalveolar Lavage (BAL), Biopsy); Systemic (Plasma or Serum)	Neutrophils; Macrophages; Eosinophils; Lymphocytes (White Blood Cells).
Biochemical Measures – Local (Exhaled, Sputum, BAL, Biopsy); Systemic (Plasma or Serum)	Leukotriene B4 (LTB4); 8-Isoprostane (8IPT); Elastase; Myeloperoxidase (MPO); Secretory Leukoprotease Inhibitor (SLPI); Endothelin-1 (ET-1); Interleukin-8 (IL8); Interleukin-6 (IL6); Interleukin-10 (IL10); Nitric Oxide; Tumour Necrosis Factor (TNFα); C-Reactive Protein (CRP); Fibrinogen.

Many study variables were measured at or around the time of the exacerbation. If these variables were measured in the stable condition of these COPD patients, i.e., measurements were taken weeks or months prior to the exacerbation, then these were also obtained.

We were also aware of the possibility that for some study groups in severity Levels II and III (as per the ATS/ERS criteria) included in this review may have experienced a similar quality of care or medical management that was not reported adequately in the original publication. In attempt to correct for this problem, we combined the exacerbation data from Levels II and III into an 'in-patient' category and then compared it to Level I that we regarded as the 'out-patient' category.

### Statistical methods

We collected and calculated study means, medians, standard errors, standard deviations, 95% confidence interval, and inter-quartile ranges using the statistical algorithms in Microsoft Excel 2002. We then conducted fixed effect meta-analyses to obtain mean point estimates, 95% confidence intervals, and two standard deviations for each exacerbation level [40]. Exacerbation Severity Levels I and II, II and III, and I and III were each compared using a two-tailed Z-test. The alpha level of p < 0.05 was adjusted for multiple testing according to the Bonferroni correction procedure [41]. In the event that a specific exacerbation severity level had a large number of studies in which only median data were available, the data were considered to be normally distributed and medians were treated as means. Since many studies did not publish data for individual patients, we were limited in addressing non-normality in the data by using a log_10_-transformation.

We again performed a fixed effect meta-analysis to obtain mean point estimates, 95% confidence intervals, and two standard deviations for in-patient and out-patient categories of each measure. We then compared each category using a two-tailed Z-test and a p-value of 0.05.

## Results

### Description of studies and study subjects

Our search strategy yielded 268 suitable studies that met our selection criteria. These studies were published between 1979 and July 2004 – Week 2. (The references for these studies can be found at the LACDR Division of Pharmacology website [42]). The total number of study subjects included in this review was 142,407. Of this group, 18% fell in Exacerbation Severity Level I, 78% in Level II, and 4% in Level III. When we re-analysed the data according to out- or in-patient settings, 18% were out-patients and 82% in-patients.

Meta-analyses of typical study demographics showed that there was significant overlap in 95% confidence intervals and study data distributions for the three exacerbation severity levels except for age where study patients in Level II had a mean age of 64.2 years (95% confidence interval (CI): 62.9 to 65.5 years) compared to 68.0 years (95% CI: 65.9 to 70.1 years) for patients in Level III (p = 0.002) (Table 2). When the demographics were re-analyzed according to patient settings, we determined that only body mass index was statistically different between the out-patient setting (mean point estimate: 26.2 kg/m^2^; 95% CI: 23.8 to 28.7 kg/m^2^) and the in-patient setting (mean point estimate: 23.4 kg/m^2^; 95% CI: 22.5 to 24.3 kg/m^2^) (p = 0.038) (Table 3).

**Table 2:** Typical Subject Demographics According to ATS/ERS 2004 Exacerbation Severity Level

**Variable/Exacerbation Severity Level**^†^	Total Studies*	**Total Subjects**^#^	**Point Estimate (95% CI)**	**Study Data Distributions (± 2SD)**	**Significance Test**^‡^	
					
					**Z Test**	**P Value**
**Age (years)**	**182**	**27,930**			Levels	
Level I	71	16,917	66.0 (64.6 – 67.4)	51.2 – 80.8	I vs. II	0.067 (NS)
Level II	70	7,300	64.2 (62.9 – 65.5)	52.1 – 76.3	II vs. III	0.002
Level III	43	3,713	68.0 (65.9 – 70.1)	53.0 – 83.0	I vs. III	0.12 (NS)

**Height (cm)**	**27**	**3,154**			Levels	
Level I	14	2,581	169.7 (166.4 – 173.0)	155.1 – 184.3	I vs. II	0.25 (NS)
Level II	9	247	167.1 (164.0 – 170.2)	157.5 – 176.6	II vs. III	0.54 (NS)
Level III	4	326	169.1 (163.4 – 174.7)	157.3 – 180.9	I vs. III	0.85 (NS)

**Weight (kg)**	**37**	**4,168**			Levels	
Level I	15	3,176	72.8 (67.5 – 78.1)	46.9 – 98.7	I vs. II	0.36 (NS)
Level II	12	290	69.3 (63.8 – 74.7)	49.4 – 89.1	II vs. III	0.26 (NS)
Level III	10	702	63.6 (55.3 – 71.9)	35.5 – 91.7	I vs. III	0.067 (NS)

**Body-Mass Index (kg/m**^2^**)**	**29**	**4,250**			Levels	
Level I	6	2,273	26.2 (23.8 – 28.7)	18.5 – 34.0	I vs. II	0.037 (NS)
Level II	19	1,729	23.4 (22.4 – 24.4)	19.5 – 27.3	II vs. III	0.84 (NS)
Level III	7	248	23.6 (21.2 – 26.0)	17.1 – 30.1	I vs. III	0.14 (NS)

**Disease Years**	**21**	**8,606**			Levels	
Level I	15	8,354	10.9 (7.8 – 14.1)	0 – 26.4	I vs. II	0.65 (NS)
Level II	5	228	12.9 (5.0 – 20.8)	0 – 30.2	II vs. III	0.67 (NS)
Level III	2	24	16.2 (3.9 – 28.4)	0 – 33.5	I vs. III	0.42 (NS)

Symbols and Abbreviations: *Bold numbers indicate total studies (without duplicates) for the specific variable of interest; # Bold numbers indicate total subjects for all COPD exacerbation severity levels with respect to the specific variable of interest; **† **Exacerbation severity levels are based on the following ATS/ERS 2004 operational classification scheme: Level I – treated at home; Level II – requires hospitalisation ; and Level III – leads to respiratory failure; ‡ Exacerbation Levels II and III were each compared to Level I using a two-tailed Z-test in which the alpha level was adjusted according to the Bonferroni Correction procedure to account for multiple testing; and NS = Non-significant difference.

**Table 3:** Typical Subject Demographics According to Out-Patient and In-patient Settings

**Variable/Patient Setting Type**^†^	Total Studies*	**Total Subjects**^#^	**Point Estimate (95% CI)**	**Study Data Distributions (± 2SD)**	**Significance Test^‡^**	
					
					**Z Value**	**P Value**
**Age (years)**	**182**	**27,930**			0.78	0.44 (NS)
Out-patient	71	16,917	66.0 (64.6 – 67.4)	51.2 – 80.8		
In-patient	112	11,013	65.3 (64.2 – 66.4)	52.3 – 78.3		

**Height (cm)**	**27**	**3,154**			1.00	0.32 (NS)
Out-patient	14	2,581	169.7 (166.4 – 173.0)	155.1 – 184.3		
In-patient	13	573	167.5 (164.8 – 170.2)	157.5 – 177.6		

**Weight (kg)**	**37**	**4,168**			1.48	0.14 (NS)
Out-patient	15	3,176	72.8 (67.5 – 78.1)	46.9 – 98.7		
In-patient	22	992	67.5 (62.9 – 72.1)	45.1 – 89.9		

**Body-Mass Index (kg/m**^2^**)**	**29**	**4,250**			2.08	0.038
Out-patient	6	2,273	26.2 (23.8 – 28.7)	18.5 – 34.0		
In-patient	24	1,977	23.4 (22.5 – 24.3)	19.1 – 27.7		

**Disease Years**	**21**	**8,606**			0.78	0.44 (NS)
Out-patient	15	8,354	10.9 (7.8 – 14.1)	0 – 26.4		
In-patient	6	252	13.9 (7.1 – 20.7)	0 – 31.7		

Symbols and Abbreviations: *Bold numbers indicate total studies (without duplicates) for the specific variable of interest; # Bold numbers indicate total subjects for out-patient and in-patient categories with respect to the specific variable of interest; **† **Out-patient category represents ATS/ERS 2004 Exacerbation Severity Level I (treated at home) and the in-patient category Levels II (requires hospitalisation) and III (leads to respiratory failure) combined; ‡ Outpatient and in-patient categories were compared using a two-tailed Z-test; and NS = Non-significant difference.

### Relationship of different variables and biomarkers with exacerbation severity and patient setting

**Figure 1:**
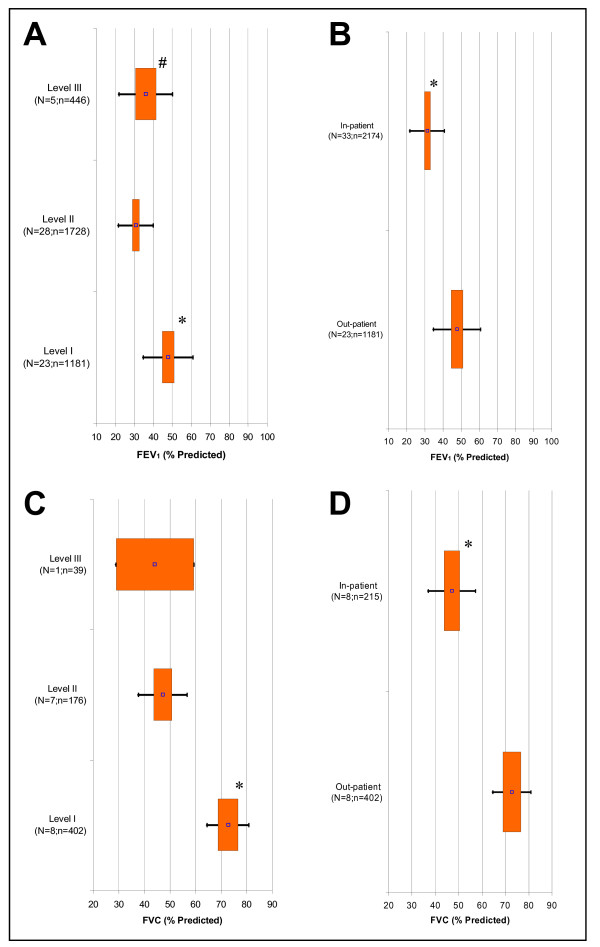
**Fixed Effect Meta-Analysis Results of Selected Spirometry Variables. **Graphs displayed are: A) FEV_1 _% Predicted (COPD Exacerbation Severity Levels I to III); B) FEV_1 _% Predicted (Out- versus In-patient Setting); C) FVC % Predicted (Levels I to III); and D) FVC % Predicted (Out- versus In-patient Setting). For each spirometry variable, the point estimates (point), 95% confidence intervals (box), and two standard deviations (bars) are presented for Levels I to III and out- & in-patient settings. 'N' signifies the total studies and 'n' is the total subjects. P < 0.017 is indicated for statistical comparisons of Level I versus II (*), II versus III (**†**), and I versus III (#) as well as P < 0.05 for comparison of out- versus in-patient setting (*).

**Figure 2:**
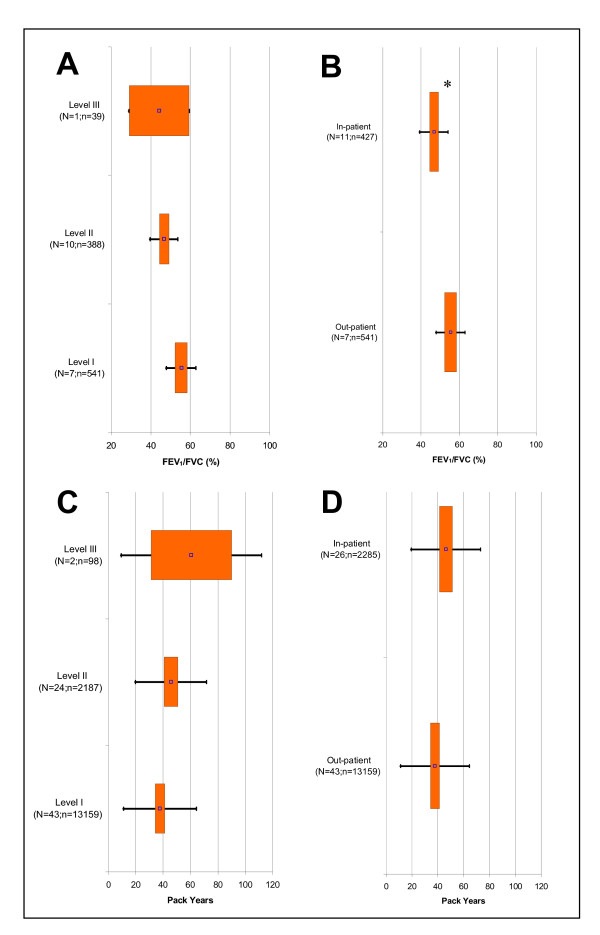
**Fixed Effect Meta-Analysis Results of Selected Clinical Variables. **Graphs displayed are: A) FEV_1_/FVC Ratio (Exacerbation Severity Levels I to III); B) FEV_1_/FVC Ratio (Out- versus In-patient Setting); C) Pack Years (Levels I to III); and D) Pack Years (Out- versus In-patient Setting). For each clinical variable, the point estimates (point), 95% confidence intervals (box), and two standard deviations (bars) are presented for Levels I to III and out- & in-patient settings. 'N' signifies the total studies and 'n' is the total subjects. P < 0.017 is indicated for statistical comparisons of Level I versus II (*), II versus III (**†**), and I versus III (#) as well as P < 0.05 for comparison of out- versus in-patient setting (*).

The spirometry measures Forced Expired Volume in 1 Second (FEV_1_) and Forced Vital Capacity (FVC), both in percent predicted, decreased from Exacerbation Levels I to II (p < 0.017) but remained unchanged from Levels II to III (Figure 1A and 1C, respectively). However, when Levels II and III were combined to create an 'in-patient' category for each of these variables, there was a statistically significant decrease for the in-patients versus the out-patients (p < 0.05) (Figure 1B and 1D, respectively). We also observed the same trend for FEV_1_/FVC (Figure 2A and 2B). For all other spirometry measures, there were too few studies available in Level III for meta-analysis.

We found for smoking that pack years increased with exacerbation severity, but only Levels I and II were statistically different (p = 0.015) (Figure 2C). When we compared pack years between patient settings, it was statistically higher for the in-patients than the out-patients (p = 0.010) (Figure 2D).

**Figure 3:**
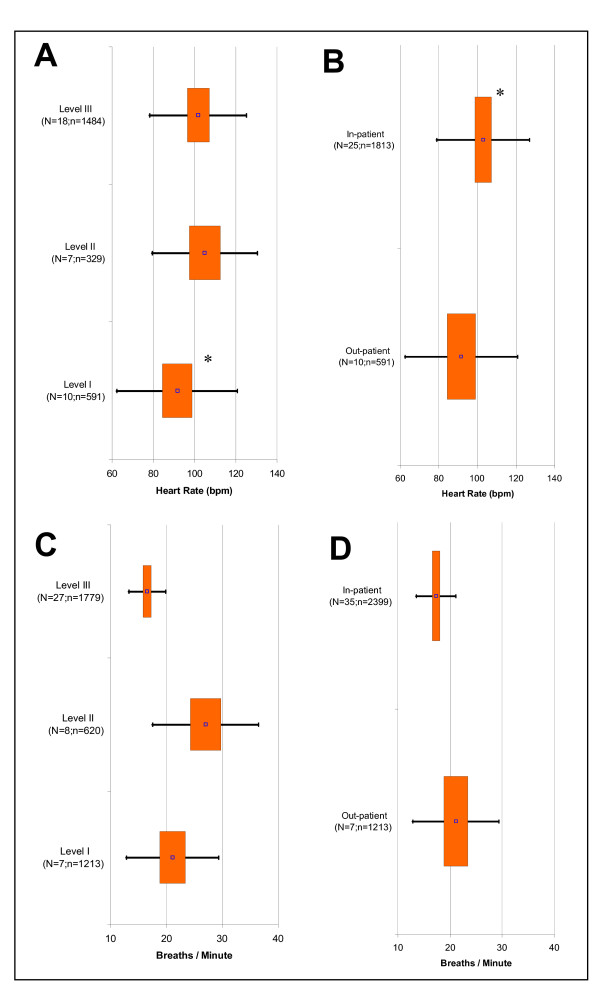
**Fixed Effect Meta-Analysis Results of Selected Clinical Variables. **Graphs displayed are: A) Heart Rate (Exacerbation Severity Levels I to III); B) Heart Rate (Out- versus In-patient Setting); C) Breathing Rate (Levels I to III); and D) Breathing Rate (Out- versus In-patient Setting). For each clinical variable, the point estimates (point), 95% confidence intervals (box), and two standard deviations (bars) are presented for Levels I to III and out- & in-patient settings. 'N' signifies the total studies and 'n' is the total subjects. P < 0.017 is indicated for statistical comparisons of Level I versus II (*), II versus III (**†**), and I versus III (#) as well as P < 0.05 for comparison of out- versus in-patient setting (*).

In terms of the hemodynamic measures, only heart rate showed a statistically significant difference being higher in Level II than Level I (p = 0.014) with no difference between Levels II and III (Figure 3A). Heart rates were also higher for in-patients than out-patients (p = 0.011) (Figure 3B).

The clinical measures of dyspnoea, i.e., the breathing rate (Figure 3C) and Borg dyspnoea score, tended to increase from Levels I to II and then decrease from Levels II to III. However, only breathing rate demonstrated clear statistical differences between the three levels (p < 0.017). Only Levels II and III of the Borg Dyspnoea Score were statistically different (p < 0.001); a statistical comparison of these levels with Level I was not possible due to lack of data. When patient settings were compared, only breathing rate showed a clear statistical difference being statistically lower for in-patients than out-patients (p = 0.003) (Figure 3D).

Exacerbation Levels II and III were statistically different with respect to pH (p = 0.003) and bicarbonate (p = 0.002) in that pH decreased from Level II to III whereas bicarbonate increased. However, there was insufficient Level I data for each variable to allow for statistical comparisons with the other Levels. There was also insufficient data available to compare out-patients with in-patients.

**Figure 4:**
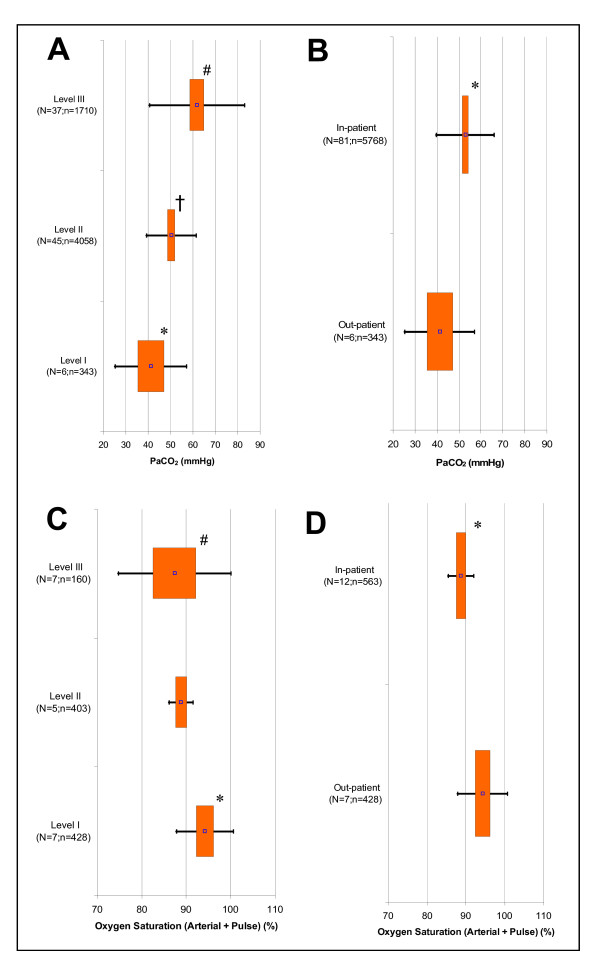
**Fixed Effect Meta-Analysis Results of Selected Clinical Variables. **Graphs displayed are: A) Arterial Carbon Dioxide Tension, PaCO_2_(Exacerbation Severity Levels I to III); B) PaCO_2 _(Out- versus In-patient Setting); C) Percent Oxygen Saturation – Arterial & Pulse Measurements Combined (Levels I to III); and D) Percent Oxygen Saturation – Arterial & Pulse Measurements Combined (Out- versus In-patient Setting). For each clinical variable, the point estimates (point), 95% confidence intervals (box), and two standard deviations (bars) are presented for Levels I to III and out- & in-patient settings. 'N' signifies the total studies and 'n' is the total subjects. P < 0.017 is indicated for statistical comparisons of Level I versus II (*), II versus III (**†**), and I versus III (#) as well as P < 0.05 for comparison of out- versus in-patient setting (*).

In terms of blood gas measures studied, only arterial carbon dioxide tension (PaCO_2_) showed a statistically significant increase with increasing exacerbation severity (p < 0.017) (Figure 4A) as well as out- versus in-patients (p < 0.05) (Figure 4B). In the case of oxygen saturation, it gradually decreased with increasing exacerbation severity with statistically significant differences between Levels I and II (p < 0.001) as well as Levels I and III (p = 0.011) (Figure 4C). It also decreased going from an out-patient to an in-patient setting (P < 0.001) (Figure 4D).

The six minute walking distance challenge test seemed to show a decreasing trend with increasing exacerbation severity but such changes did not reach statistical significance. This was also the case when the out- and in-patients were compared. Many other variables related to spirometry, respiratory status, exacerbation and hospital event categories also did not change significantly with exacerbation severity or out- and in-patients (See additional file: 1). There was not enough data in the bacteriology and virology categories to permit any meta-analyses. Of the 268 studies sampled, only half contained data about the biochemical variables.

## Discussion

We conducted this review of the COPD exacerbation literature to determine which commonly-accepted baseline variables and suggested markers changed in a consistent manner with the severity of COPD exacerbations. As our index of COPD severity, we used the recently published ATS/ERS operational classification of exacerbation severity for medical management. This is because most of the published literature rarely provides sufficient details to characterise the severity of a patient's exacerbation. In addition, we also analyzed the same data according to out- and in-patient settings so as to account for possible overlaps in medical management between Levels II and III but were not reported in the original publication.

The long-term aim of our work is to improve the quality and applicability of exacerbation management through the identification of sensitive and specific markers that can be used for the assessment of treatment effects. This review identified a few potential markers of exacerbation severity.

When we assessed the spirometry measures FEV_1 _and FVC in % predicted, as well as FEV_1_/FVC, we observed statistically significant differences with exacerbation severity, and between out- and in-patients (Figures 1A–D and 2A–B). One draw-back was the paucity of such information in Level III studies. This confirms the clinical situation that as exacerbations worsen and more specialised care is required, spirometry measurements are less likely under baseline conditions or during an exacerbation [14]. Thus, such data is rare in many published studies.

The number of smoking-related pack years increased with exacerbation severity and showed a clear difference between out- and in-patient settings (Figures 2C and 2D), a finding that is consistent with the idea that the more a COPD patient smokes, and for longer, the higher the likelihood that COPD exacerbations will be more severe. According to the mean point estimates obtained in this study, COPD patients with 40 to 60 pack-years of smoking will experience an increase in the severity of COPD exacerbations. However, our conclusion regarding this finding is limited by there being data from only two studies at Level III.

Although heart rate varied little between Exacerbation Levels II to III, it is important to note that it was substantially elevated in patients (Figure 3A) with the clearest difference being between in- and out-patients. This is possibly associated with the anxiety and dyspnea that experienced when an exacerbation occurs. The increase in heart rate of course increases the oxygen requirements of the heart. The increased heart rate may also be the result of underlying cardiovascular disease that is more prominent in severe COPD patients [43].

The relationship of pH and bicarbonate to exacerbation severity are consistent with the signs of respiratory acidosis evident in COPD patients with exacerbations [1,24,25]. However, due to the shortage of data in Level I, proper statistical conclusions about each of these variables are difficult to make. In relation to this, breathing rate significantly increased from Levels I to II and then decreased from Levels II to III (Figure 3C). The first observation may reflect components of the exacerbation episode (i.e., anxiety and dyspnea) as well as the physiological need to breathe more to maintain adequate blood gas levels. The reduction at Level III possibly reflects the results of the specialized care where patients are given ventilatory support so as to return the breathing rate to normal. The Borg Dyspnea Score showed the same trend as breathing rate, although insufficient data in Level I did not allow for further comparisons. When out- and in-patient data were compared for each of these variables, only breathing rate demonstrated a clear statistical difference (Figure 3D). The Borg Dyspnoea Score on the other hand did not have enough studies in the out-patient category to perform any statistical test. Overall, the observed trends were consistent with the fact that management of dyspnoea is one of the main factors generating the high hospital costs associated with COPD exacerbations [44]. In keeping with the direct measures of dyspnoea, arterial carbon dioxide tension showed a clear relationship with exacerbation severity and patient management settings (Figures 4A and 4B) that is consistent with the conclusions reported in the medical literature [20,45-47]. Arterial oxygen tension in contrast did not change with exacerbation severity or patient setting. Possibly this lack of correlation reflects the immediate administration of supplemental oxygen given to hypoxaemic patients in a hospital setting. There was however a decreasing trend in oxygen saturation with increasing exacerbation severity and clear differences between out- and in-patient settings (Figures 4C and 4D) that are consistent with the present thinking on blood gas changes.

Most of the other commonly accepted measures and suggested biomarkers poorly reflected exacerbation severity, or the fact that there was not sufficient data to undertake a meta-analysis (See additional file: 1). This finding recalls a 2001 US Department of Health and Human Services report on exacerbation treatment outcomes from over 200 randomised controlled trials [14]. The aim of that study was to create new guidelines to improve the management of COPD exacerbations. That study also concluded that the current literature was limited in terms of the number of studies and the amount of detail available as well as the reliability and accuracy of the clinical assessments used to discriminate between COPD exacerbations and other causes of worsening respiratory status. Thus, our observations agree with previous observations regarding the assessment of the unstable COPD literature.

As previously discussed, most of the studies used for this review were predominately with hospitalized patients (Level II). However, most COPD occurs in an out-patient setting (Level I) [48-53]. This has implications for our study since the latter population was poorly represented.

Our basic categorisation was according to the ATS/ERS' operational scheme for classifying the severity of COPD exacerbations as well as to out- and in-patient categories. To our knowledge, we are the first to undertake this type of literature review and thus we were faced with a lack of consistency in the definition of exacerbations as used in the various studies. We tried to overcome this difficulty by selecting and ranking clinical studies so as to improve the comparability of subjects between studies.

We were also aware that the clinical studies we analysed differed with respect to which comorbidities or identifiable causes for exacerbations were reported. Most patients were elderly and therefore were more likely to be suffering from one or more co-existing diseases such as asthma or cardiovascular disease. Such co-morbidity makes interpretation of our findings more difficult with respect to the true causes of exacerbations. If their aetiology could be determined, then susceptible patients such as those in Level I could be identified and new treatments developed to help prevent their onset and related hospital costs.

Finally, the compatibility between the studies of COPD exacerbation that we analysed may have been limited by substantial variations in the time and location of studies. Exacerbations are more likely in summer [5] but many studies failed to report the time of year or the time period for study implementation. Thus, seasonal effects, combined with the low incidence of exacerbations per patient, could represent an inherent bias. In addition, different institutions probably had different standards with respect to diagnosis and management of COPD exacerbations when these studies were performed. Such variations may also explain any observed inconsistencies in our findings. However, we attempted to overcome this possible bias in Exacerbation Levels II and III by the subsequent re-analysis of this data on the basis of out-patient and in-patient settings.

As observed in The additional online file, there was a scarcity of information particularly for biomarkers at different exacerbation levels. It is also unclear to us whether any of the variables that changed with exacerbation severity are causally-related. Hence, longitudinal studies and/or less restrictive eligibility criteria would be needed to address all these questions. One difficulty in tackling such problems is the enormous amount of time and expense involved in implementing such studies. In addition, the current methods for data analysis in clinical studies have limitations imposed by the assessment of the reduction in frequency or total suppression of exacerbation episodes (i.e. rare event or "non-event").

To overcome these drawbacks and obtain more accurate evaluation of treatment effect on COPD exacerbations, alternative analytical methods based, for example, on predictive mathematical models such as hidden Markov chains or Bayesian forecasting should be tried. Such models can characterise and predict rare events without undertaking a full-scale, long-term longitudinal study. This approach to predicting rare events has been used previously in studies of migraine, epilepsy and various cardiovascular diseases where the size of treatment effect is measured in terms of a reduction in the frequency of the repetition of an event within a given probability or within a given time period [54,55]. One example of a mathematical model development includes the use of a Markov model to predict COPD exacerbation rates in a clinical trial of the inhaled anticholinergic bronchodilator tiotropium [56]. In this example, the model was developed on the basis of prior knowledge of the exacerbation rate as estimated from meta-analyses of randomised controlled trial data. This gave the probabilities for COPD exacerbations for different stages of COPD. In another study, a proportional hazards model was used to identify risk factors for COPD patients hospitalised due to an exacerbation [44]. The current ATS/ERS guidelines for exacerbations do not consider the implications of using probabilistic models as a means of assessing the severity of COPD exacerbations or the effect of treatment [1]. A modelling approach may offer new insights into which variables related to COPD exacerbations should be investigated.

From a research planning perspective, our study findings have generated some hypotheses and related considerations that could be evaluated in future clinical trials. One hypothesis is that the combination of variables that we observed to change in our study (i.e., FEV_1_, FVC, FEV_1_/FVC, arterial carbon dioxide, breathing rate, heart rate, pack years, and oxygen saturation) could represent a new definition for a 'severe' exacerbation event. Most definitions in the literature, including the recent ATS/ERS definition, do not indicate any assessment of (patho)physiological variables as signs of an exacerbation. They simply regard the exacerbation as a worsening of the normal day-to-day symptoms and/or an adjustment in medical management [17]. A definition that encompasses a clear set of objective measures would be useful to medical practitioners who predominantly rely on clinical judgement or past experiences for diagnosing an exacerbation and its severity as well as for assessing treatment effect.

Another important consideration for future clinical trials is the assessment of treatment effect based on predictions of exacerbation frequency and intensity. In other words, the collection of data such as the rate of onset and resolution of an exacerbation from longitudinal studies could be used to determine probabilities of second, third, fourth, etc., exacerbation events in individual patients [54]. The alteration of such probabilities with an experimental treatment could be a more sensitive and reliable approach for assessing treatment effect in clinical trials than recording daily changes in symptoms or medical management.

Lastly, our findings were obtained from COPD patients that had experienced at least one exacerbation during the study assessment period. In the same studies, there were also patients who did not experience an exacerbation. This indicates that a fraction of COPD patients may be regarded as being susceptible to an exacerbation whereas another fraction is 'exacerbation-free'. It would be interesting to determine how the variables we identified in our study change in the latter patient group according to FEV_1_. Some published studies have stratified COPD patients on the basis of exacerbation frequency; this is generally done by categorising patients as having either 'infrequent' or 'frequent' exacerbations if they had less than or greater than a mean of three exacerbations per year, respectively [57]. In our study, we were unable to make this distinction between COPD patients since many of the published studies did not provide individual patient data on exacerbation frequency. We are currently investigating a commercial database of clinical trials that will enable us to look at patients with 'infrequent' or 'frequent' exacerbations. The results of this work could help us better select patients as well as identify potential markers for future longitudinal studies.

## Conclusion

The current management and treatment of COPD exacerbations is primarily dependent on the evaluation of the symptoms rather than the signs related to the exacerbation event. We found that arterial carbon dioxide tension and breathing rate consistently varied with the severity of COPD exacerbations and with in- versus out-patients. Other commonly-accepted measures and suggested biomarkers for exacerbations failed to show consistent trends or lacked sufficient data to permit any meta-analysis. We recommend the design of longitudinal studies looking at the frequency of exacerbations as well as the use of more advanced modelling techniques to improve the selection of potential markers for the categorization of the severity of COPD exacerbations and the assessment of treatment effect in future studies.

## Abbreviations

ATS – American Thoracic Society

ERS – European Respiratory Society

COPD – Chronic Obstructive Pulmonary Disease

FEV_1 _– Forced Expired Volume in One Second

FVC – Forced Vital Capacity

## Competing interests

The author(s) declare that they have no competing interests.

## Authors' contributions

LF and ODP contributed to the research concept. LF performed the data collection, abstraction and analysis. All authors contributed to data interpretation. LF wrote the first draft of the manuscript and all authors took part in the revision and final version of this report.

## Supplementary Material

Additional file 1 table: Variables That Demonstrated Little Relationship with Exacerbation Severity or Patient Setting, or Had Insufficient Data for Meta-Analysis. Click here for file
