# Supplementary material for: Markers of exacerbation severity in chronic obstructive pulmonary disease
Source: Respir Res. 2006 May 10;7(1):74. doi: 10.1186/1465-9921-7-74 (PMC1481583; doi:10.1186/1465-9921-7-74)
Supplement: Additional file 1 — table: Variables That Demonstrated Little Relationship with Exacerbation Severity or Patient Setting, or Had Insufficient Data for Meta-Analysis. [file 1465-9921-7-74-s1.doc]

**Additional file:**

**table: Variables That Demonstrated Little Relationship with Exacerbation Severity or Patient Setting, or Had Insufficient Data for Meta-Analysis**

| **Variable** | **Total Studies** | **Total Subjects** |
| --- | --- | --- |
| **Spirometry & Respiratory Status** |  |  |
| PaO_2_ (mmHg) | 84 | 6,617 |
| PaO_2_/FiO_2_ Ratio | 18 | 1,696 |
| PEFR (L/min) | 12 | 3,069 |
| FiO_2_ (mmHg) | 12 | 715 |
| Oxygen Saturation (Arterial) (%) | 12 | 389 |
| PEEP_i_ (cmH_2_O) | 9 | 726 |
| Oxygen Saturation (Pulse) (%) | 7 | 602 |
| Sputum Production (wet weight grams) | 4 | 192 |
| **Dyspnea** |  |  |
| Borg Dyspnea Score (Total Score/10) | 6 | 743 |
| Baseline Dyspnea Index (BDI) | 2 | 1,233 |
| Transitional Dyspnea Index (TDI) | 2 | 230 |
| Medical Research Council (MRC) Dyspnea Scale | 2 | 144 |
| American Thoracic Society (ATS) Dyspnea Score | 1 | 135 |
| **Functional Challenge & Quality of Life** |  |  |
| Beta Agonist Reversibility (% FEV_1_ Predicted) | 14 | 4,238 |
| St. George’s Resp. Questionnaire (Total Score/100) | 13 | 4,552 |
| 6 Minute Walking Distance (metres) | 7 | 1,649 |
| Adenosine Monophosphate Challenge | N.Av. | N.Av. |
| Histamine Challenge | N.Av. | N.Av. |
| Methacholine Challenge | N.Av. | N.Av. |
| **Haemodynamic** |  |  |
| Systolic Blood Pressure (mmHg) | 13 | 637 |
| Diastolic Blood Pressure (mmHg) | 10 | 550 |
| Mean Arterial Blood Pressure (mmHg) | 7 | 1,166 |
| Cardiac Output (L/minute) | 7 | 104 |
| **Electrocardiogram** |  |  |
| Electrocardiogram – Lead II | 1 | 50 |
| Electrocardiogram – Lead aVF | 1 | 50 |
| Electrocardiogram – P Wave Axis (degrees) | 1 | 50 |

**TABLE: Variables That Demonstrated Little Relationship With Exacerbation Severity or Patient Setting, or Had Insufficient Data for Meta-Analysis** (Continued)

| **Variable** | **Total Studies** | **Total Subjects** |
| --- | --- | --- |
| **Blood pH, Electrolyte or Protein** |  |  |
| pH | 62 | 4,612 |
| Bicarbonate (mmole/L) | 15 | 757 |
| Albumin (mmole/L) | 8 | 864 |
| Potassium (millimole/L) | 6 | 369 |
| Urea (millimole/L) | 5 | 253 |
| Creatinine (micromole/L) | 4 | 630 |
| Sodium (millimole/L) | 4 | 183 |
| Glucose (millimole/L) | 2 | 216 |
| Phosphate (millimole/L) | 2 | 55 |
| Chloride (millimole/L) | 1 | 49 |
| **Exacerbation Event Related** |  |  |
| Days in Hospital | 47 | 81,237 |
| No. of Exacerbations Per Year (or Past Year) | 16 | 6,003 |
| No. of Exacerbations Per Patient-Year | 11 | 2,947 |
| Time to First Exacerbation | 11 | 1,425 |
| Number of Unscheduled GP Visits in Past Year | 3 | 3,195 |
| Days Per Patient-Year in Hospital | 2 | 771 |
| No. of Exacerbation Infections in Past Year | 2 | 711 |
| No. of Exacerbation Oral Steroids Per Patient-Year | 2 | 617 |
| Number of Scheduled GP Visits in Past Year | 1 | 400 |
| **Hospital Related** |  |  |
| Number of Patients Hospitalized in Past Year | 14 | 6,384 |
| Number of Patients Re-admitted in Past Year | 6 | 842 |
| Number of Emergency Dept. Visits in Past Year | 4 | 3,125 |
| Number of Patients Relapsed in Past Year | 4 | 1,648 |
| Number of Admissions in a Year | 4 | 606 |
| Number of Admissions Per Patient-Year | 3 | 1,458 |
| Admissions to the Intensive Care Unit | 3 | 1,081 |

**table: Variables That Demonstrated Little Relationship With Exacerbation Severity or Patient Setting, or Had Insufficient Data for Meta-Analysis (Continued)**

| **Variable** | **Total Studies** | **Total Subjects** |
| --- | --- | --- |
| **Bacteriology** |  |  |
| Number of Patients & Isolate Assessments of S. Pneumoniae; H. Influenzae; M. Catarrhalis; P. Aeruginosa; B. Catarrhalis; H. Parainfluenza; S. Aureus; C. Pneumoniae; E. Coli; . Pneumoniae, Enterobacteriaceae, Pseudomonas Species, Alpha-Haemolytic Streptococci, Acinetobacter, M. Pneumoniae, Legionella Species. | 37 | 5,473 |
| **Virology** |  |  |
| Number of Patients & Isolate Assessments of Influenza Virus A & B; Parainfluenza V1, V2 & V3; Adenovirus; Respiratory Syncytial Virus (RSV); Picornavirus; Rhinovirus; Coronavirus. | 4 | 458 |
| **Cytology** |  |  |
| Blood Lymphocytes (x10^9^/L) | 11 | 967 |
| Sputum Neutrophils (% of Differential Count) | 6 | 236 |
| Sputum Macrophages (% of Differential Count) | 4 | 236 |
| Blood Eosinophils (x10^9^/L) | 4 | 90 |
| Sputum Eosinophils (% of Differential Count) | 4 | 65 |
| Sputum Lymphocytes (% of Differential Count) | 3 | 57 |
| Biopsy Neutrophils (cells/mm^2^) | 2 | 20 |
| Biopsy Macrophages (cells/mm^2^) | 2 | 20 |
| Biopsy Eosinophils (cells/mm^2^) | 2 | 20 |
| Blood Neutrophils (x10^9^/L) | 1 | 43 |
| Biopsy Lymphocytes (cells/mm^2^) | 1 | 9 |
| BAL Neutrophils (% of Differential Count) | 1 | 6 |
| BAL Macrophages (% of Differential Count) | 1 | 6 |
| BAL Eosinophils (% of Differential Count) | 1 | 6 |
| BAL Lymphocytes (% of Differential Count) | 1 | 6 |
| Blood Macrophages | N.Av. | N.Av. |

**Table: Variables That Demonstrated Little Relationship With Exacerbation Severity or Patient Setting, or Had Insufficient Data for Meta-Analysis (Continued)**

| **Variable** | **Total Studies** | **Total Subjects** |
| --- | --- | --- |
| **Biochemical** |  |  |
| Blood C-Reactive Protein (mg/L) | 11 | 431 |
| Sputum Interleukin-8 (nanograms/mL) | 7 | 186 |
| Blood Interleukin-6 (picograms/mL) | 3 | 111 |
| Sputum Leukotriene B4 (nM) | 3 | 99 |
| Sputum Myeloperoxidase (Units/mL) | 3 | 99 |
| Sputum Interleukin-6 (picograms/mL) | 3 | 73 |
| Blood Interleukin-8 (picograms/mL) | 2 | 54 |
| Sputum Elastase (nM) | 2 | 30 |
| Exhaled Nitric Oxide (parts per billion) | 2 | 29 |
| Blood Endothelin-1 (picograms/mL) | 2 | 27 |
| Blood Fibrinogen (grams/L) | 1 | 67 |
| Blood Myeloperoxidase (micrograms/L) | 1 | 43 |
| Blood Interleukin-10 (picograms/mL) | 1 | 34 |
| Sputum Secretory Leukoprotease Inhibitor (uM) | 1 | 22 |
| Exhaled Leukotriene B4 (picograms/mL) | 1 | 21 |
| Exhaled 8-Isoprostane (picograms/mL) | 1 | 21 |
| Blood TNF-Alpha (picograms/mL) | 1 | 16 |
| Sputum TNF-Alpha (picograms/mL) | 1 | 14 |
| Sputum Endothelin-1 (picograms/mL) | 1 | 14 |
| Biopsy TNF-Alpha (TNF+ve cells/mm^2^) | 1 | 11 |
| Blood Leukotriene B4 (picograms/mL) | 1 | 8 |
| Exhaled Carbon Monoxide | N.Av. | N.Av. |

**Table: Variables That Demonstrated Little Relationship With Exacerbation Severity or Patient Setting, or Had Insufficient Data for Meta-Analysis (Continued)**

| **Variable** | **Total Studies** | **Total Subjects** |
| --- | --- | --- |
| **Biochemical (Continued)** |  |  |
| Sputum 8-Isoprostane | N.Av. | N.Av. |
| Sputum C-Reactive Protein | N.Av. | N.Av. |
| Sputum Fibrinogen | N.Av. | N.Av. |
| Sputum Interleukin-10 | N.Av. | N.Av. |
| BAL 8-Isoprostane | N.Av. | N.Av. |
| BAL C-Reactive Protein | N.Av. | N.Av. |
| BAL Elastase | N.Av. | N.Av. |
| BAL Endothelin-1 | N.Av. | N.Av. |
| BAL Fibrinogen | N.Av. | N.Av. |
| BAL Interleukin-6 | N.Av. | N.Av. |
| BAL Interleukin-8 | N.Av. | N.Av. |
| BAL Interleukin-10 | N.Av. | N.Av. |
| BAL Leukotriene B4 | N.Av. | N.Av. |
| BAL Myeloperoxidase | N.Av. | N.Av. |
| BAL Secretory Leukoprotease Inhibitor | N.Av. | N.Av. |
| BAL TNF-Alpha | N.Av. | N.Av. |
| Biopsy 8-Isoprostane | N.Av. | N.Av. |
| Biopsy C-Reactive Protein | N.Av. | N.Av. |
| Biopsy Elastase | N.Av. | N.Av. |
| Biopsy Endothelin-1 | N.Av. | N.Av. |
| Biopsy Fibrinogen | N.Av. | N.Av. |
| Biopsy Interleukin-6 | N.Av. | N.Av. |
| Biopsy Interleukin-8 | N.Av. | N.Av. |
| Biopsy Interleukin-10 | N.Av. | N.Av. |
| Biopsy Leukotriene B4 | N.Av. | N.Av. |
| Biopsy Myeloperoxidase | N.Av. | N.Av. |
| Biopsy Secretory Leukoprotease Inhibitor | N.Av. | N.Av. |
| Blood 8-Isoprostane | N.Av. | N.Av. |
| Blood Elastase | N.Av. | N.Av. |
| Blood Secretory Leukoprotease Inhibitor | N.Av. | N.Av. |
